# COVID-19-Associated Cerebellitis: A Case Report and Rehabilitation Outcome

**DOI:** 10.1007/s12311-024-01721-x

**Published:** 2024-07-15

**Authors:** Roberto Tedeschi, Vincenza Amoruso, Valentina Boetto, Davide Glorioso, Lucia D’Auria, Danilo Donati

**Affiliations:** 1 Department of Biomedical and Neuromotor Sciences (DIBINEM), Alma Mater Studiorum, University of Bologna, Via Zamboni 33, Bologna, 40126 Italy; 2 Department of Geriatrics and Orthopaedics, Università Cattolica del Sacro Cuore, Rome, 00168 Italy; 3 Department of Clinical-Surgical, Diagnostic and Pediatric Sciences, University of Pavia, Pavia, 27100 Italy; 4 Neuro-Orthopedic Unit, Sol et Salus Hospital, Rimini, 47922 Italy; 5 Physical Therapy and Rehabilitation Unit, Policlinico di Modena, Modena, 41125 Italy; 6 Clinical and Experimental Medicine PhD Program, University of Modena and Reggio Emilia, Largo del Pozzo 71, Modena, 41124 Italy

**Keywords:** COVID-19, Cerebellitis, Neurological complications, Rehabilitation, Multidisciplinary approach

## Abstract

**Introduction**. The COVID-19 pandemic has brought attention to neurological complications, including cerebellitis, characterized by inflammation of the cerebellum. Despite its rare occurrence, cerebellitis has been associated with COVID-19 infection, albeit the pathogenic mechanisms remain unclear. **Case report**. We present the case of a 22-year-old male with acute onset ataxia and dysarthria during a SARS-CoV-2 infection. Diagnostic evaluations ruled out other causes, confirming cerebellitis. Treatment included steroid therapy, vitamin supplementation, physiotherapy, and intravenous immunoglobulins. Rehabilitation focused on enhancing balance, coordination, and daily activities. The patient showed significant improvement in functional abilities, with increased autonomy in daily activities and improved ambulation. Despite persistent mild symptoms, the multidisciplinary rehabilitation approach led to remarkable progress. **Conclusions**. This case underscores the importance of recognizing and managing neurological complications, such as cerebellitis, in COVID-19 patients. A comprehensive approach combining medical treatment and rehabilitation is essential for optimizing outcomes. Further research is needed to elucidate the pathogenesis and optimal management strategies for such complications.

## Introduction

From the beginning of the COVID-19 pandemic, caused by the SARS-CoV-2 virus, evidence has emerged suggesting a wide range of clinical manifestations, beyond the primary respiratory symptoms [1]. Among the neurological complications, although rare, cerebellitis has recently gained attention due to its potential association with COVID-19 infection [1, 2]. Cerebellitis, an inflammation of the cerebellum a brain structure essential for coordination, balance, and motor control remains relatively undefined in the context of COVID-19, with reported prevalence ranging from 0.1–0.5% [3]. Neurological symptoms of cerebellitis include ataxia (coordination difficulties), dysmetria (inability to judge distance or force accurately), tremors, and nystagmus (involuntary eye movements) [4–8]. The pathogenic mechanisms underlying the association between COVID-19 and cerebellitis are not entirely clear. Hypotheses include direct inflammation, where the SARS-CoV-2 virus might directly infect the cerebellum, causing inflammation and neuronal damage; an aberrant immune response to the virus, potentially harming cerebellar tissue; and thrombotic microangiopathy, as COVID-19 is associated with an increased risk of thrombosis, which could affect cerebral vessels and lead to cerebellar damage [9]. Evidence linking COVID-19 and cerebellitis primarily comes from case reports and small case series. Nonetheless, some observational studies have identified a statistically significant association between the two conditions. Prompt recognition of cerebellitis in patients with COVID-19 is crucial for appropriate management and prognosis. Brain MRI is the diagnostic tool of choice [10]. The treatment strategy focuses mainly on anti-inflammatory and supportive therapy. While the link between COVID-19 and cerebellitis requires further investigation for a complete understanding, the growing body of evidence suggests a possible connection between the two conditions. Early identification and management of cerebellitis can enhance the clinical outcomes for patients affected by COVID-19. The connection between COVID-19 and cerebellitis, although rare, is an important area of research with implications for diagnosing, treating, and prognosticating in COVID-19 patients [11]. Future studies should aim at epidemiological and clinical investigations to more accurately determine the incidence, pathogenic mechanisms, and treatment options for this uncommon complication.

## Case Presentations

A 22-year-old male patient, non-athletic, with a history of metabolic dyslipidemia, obesity, hyperhomocysteinemia, learning disabilities, cognitive impairment, short stature, subclinical hypothyroidism, keratosis pilaris, and recurrent respiratory infections in childhood. The patient, previously unvaccinated against SARS-CoV-2, initially presented with non-respiratory symptoms including diarrhea, malaise, and headache starting on December 30, 2021. These symptoms were soon followed by neurological manifestations. By the morning of January 1, 2022, the patient experienced a low-grade fever (37.1 °C), significant alteration in speech, and marked instability in maintaining an upright posture, with a tendency to deviate to the left side and limb weakness, presented to the Emergency Department on January 1, 2022, with:

- Difficulty maintaining an upright position.
- Postural instability and lower limb weakness.
- Dysarthria.
- Trunk instability with a tendency to lean to the left.
- Low-grade fever.
- General malaise.
- Headache.
- Diarrheal stools.

**Fig. 1:**
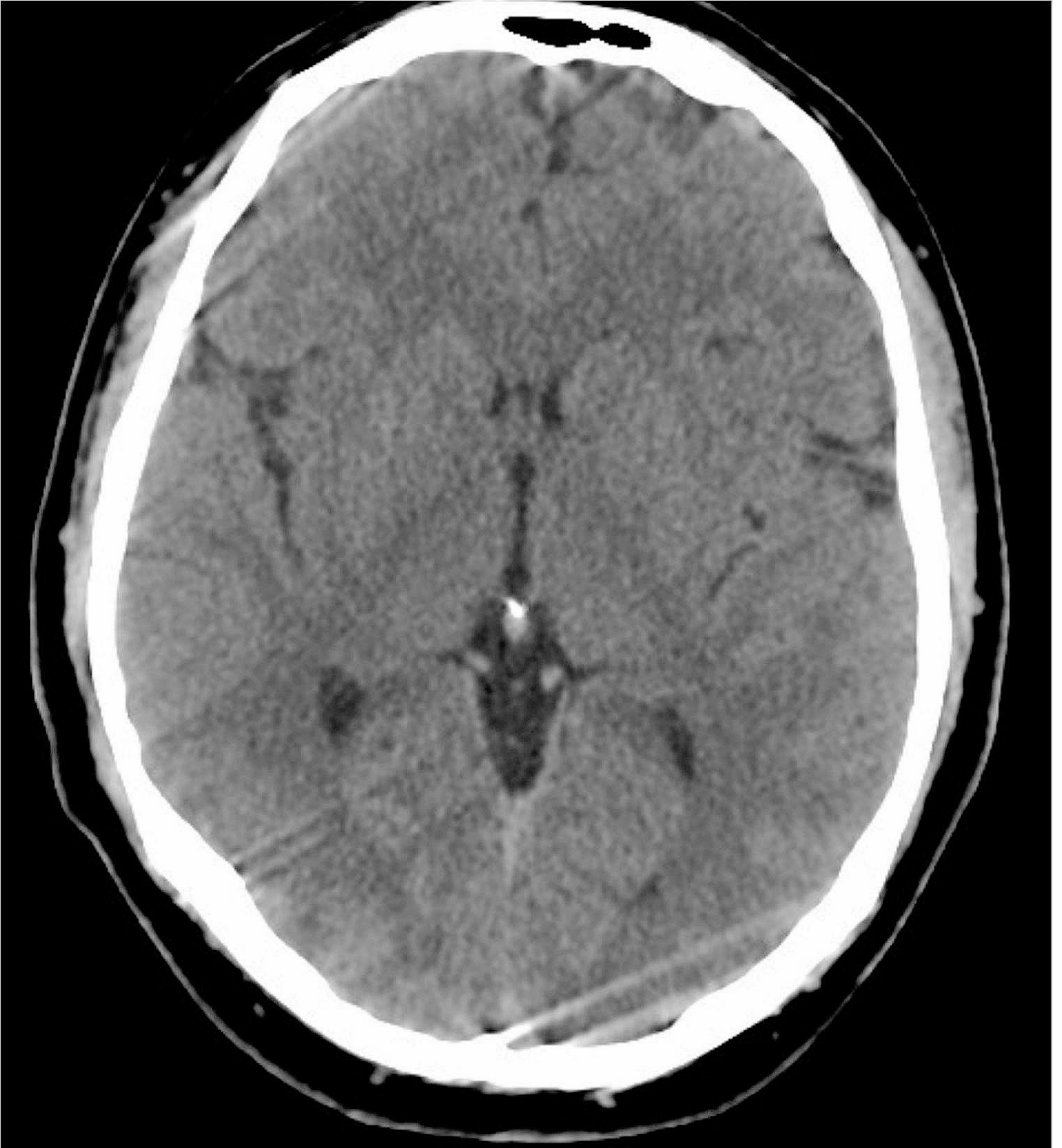
Computed Tomography (TC) Brain

**Fig. 2:**
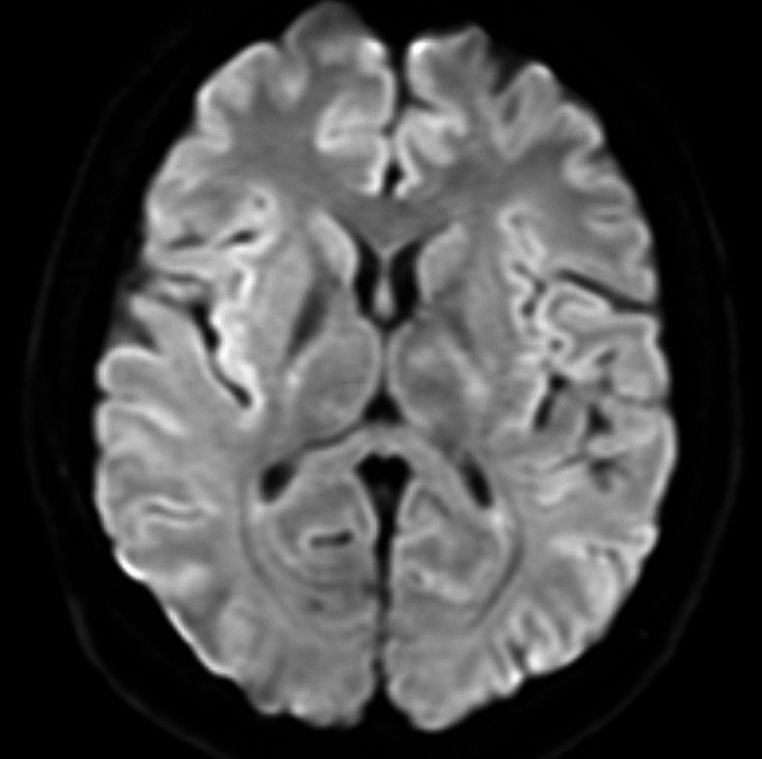
Magnetic ResonanceIimaging (RMN) Brain

**Fig. 3:**
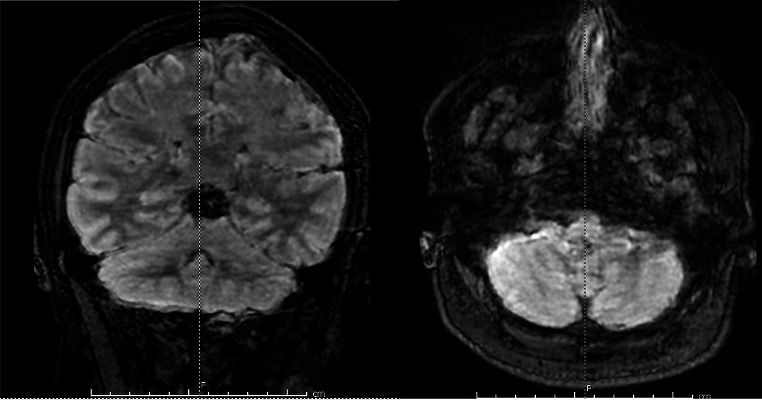
Sequences FLAIR

Diagnostic tests, including brain CT (Fig. 1) and CT angiography, brain MRI and MR angiography, lumbar puncture, two brain MRIs (Figs. 1, 2 and 3), and neurophysiological study, ruled out vascular malformations, infections, and other acute inflammatory processes. A nasopharyngeal swab for Sars-CoV-2 was positive. Upon admission, the patient underwent a lumbar puncture to obtain cerebrospinal fluid (CSF) for diagnostic analysis. Approximately 8–9 cc of CSF was extracted without complication. The fluid was clear, and initial laboratory results indicated normal chemical-physical properties. The specific results of the cerebrospinal fluid (CSF) analysis were as follows: cell count 2/μL (normal value: <5/μL), protein 35 mg/dL (normal value: 15–45 mg/dL), glucose 65 mg/dL (normal value: 40–70 mg/dL) with concurrent plasma glucose of 90 mg/dL. These results indicate a non-infectious etiology. Microbiological assays, including PCR for SARS-CoV-2 and a comprehensive panel for common neurotropic viruses, returned negative results. Tests for antibodies targeting principal intracellular and surface antigens were also negative. Investigations for the presence of Treponema Pallidum DNA were in progress at the time of report preparation.

The absence of pleocytosis, normal protein levels, and glucose in line with serum glucose—commonly assessed in CSF analyses—were indicative of a non-infectious etiology. The CSF profile, along with the absence of detectable pathogens, suggested that the cerebellar symptoms were not due to an active infection but possibly to an autoimmune/inflammatory response, potentially triggered by the recent SARS-CoV-2 infection.

The patient was admitted to the Stroke Unit and diagnosed with acute cerebellitis of probable autoimmune origin (under investigation). Initial treatment included steroid therapy, vitamin supplementation (vitamins E and B), and physical therapy (PT). Subsequently, intravenous immunoglobulins (IVIG) were administered. Several specific antibodies for cerebellitis were tested, including antibodies against major intracellular and surface antigens, all of which were negative. Specifically, no antibodies against glutamate and glutamic acid decarboxylase (GAD) were found.

The patient showed clinical stabilization, Sars-CoV-2 swabs turned negative, and he was transferred to the Rehabilitation Medicine department for ongoing rehabilitative treatment.

### Clinical Findings

The clinical findings for the 22-year-old patient presenting with acute ataxia and dysarthria amid a SARS-CoV-2 infection (Omicron variant) suggest a likely parainfectious cerebellitis. Initially, the patient faced difficulties maintaining an upright stance, postural instability, lower limb weakness, dysarthria, and a tendency for trunk instability leaning to the left, accompanied by low-grade fever, general malaise, headache, and diarrheal stools. Diagnostic procedures, including brain CT and MRI scans, lumbar puncture, and neurophysiological studies, ruled out vascular malformations, infections, and other acute inflammatory processes, with a positive nasopharyngeal swab for SARS-CoV-2. The treatment approach involved steroid therapy, vitamin supplementation, and physiotherapy [12–14], followed by intravenous immunoglobulins, leading to clinical stabilization and negative SARS-CoV-2 tests. The patient was then transferred to a rehabilitation medicine department for intensive rehabilitative treatment, focusing on regaining autonomy in standing, improving static and dynamic balance, walking autonomy, and activities of daily living (ADL). At discharge, significant improvements were noted in daily activities and walking without aids, although a slight ataxic tremor in the right hand during fine manual tasks persisted. The patient’s rehabilitation included exercises for dynamic balance improvement at home.

Coronal and Axial FLAIR MRI images demonstrate cerebellar alterations with a severity score [3] of 3, reflecting bilateral cerebellar gray matter involvement without definitive evidence of white matter alteration or contrast enhancement, and no complications observed.

### Timeline

#### January 1, 2022

The patient presents to the Emergency Department and is admitted to the Stroke Unit.

#### January 13, 2022

Treatment with steroid therapy and physiotherapy (FKT) begins; transfer to the Gastro COVID Medicine department; initiation of intravenous immunoglobulin (IVIG) administration.

#### January 18, 2022

Nasopharyngeal swabs for Sars-Cov2 turn negative.

#### February 9, 2022

The patient is transferred to the Rehabilitation Medicine department to continue intensive rehabilitative treatment.

#### March 1, 2022

The patient is discharged with significant improvements in walking and daily activities, despite a persistent slight ataxic tremor in the right hand during fine manual tasks.

### Diagnostic Assessment

The Functional Diagnostic Evaluation focuses on the functional-rehabilitative aspect for a 22-year-old male patient, assessed on March 1, 2023, diagnosed with acute onset ataxia and dysarthria during a SARS-CoV-2 infection, suggesting probable parainfectious cerebellitis. The evaluation aimed to assess the patient’s functional abilities and limitations resulting from ataxia and dysarthria, establishing functional rehabilitative goals. Significant improvements were observed in Activities of Daily Living (ADL), with the Modified Barthel Index (MBI) increasing from 65/100 to 91/100, indicating a shift from partial autonomy to autonomy. Challenges remained in dressing, feeding (swallowing difficulties), mobility (transfers, walking), personal hygiene, and household management, with prior dependency on a walker, now resolved. The evaluation also noted improved static and dynamic balance, slightly compromised coordination (right hand ataxic tremor), mild cognitive impairment in memory and phonemic fluency, and persisting mild articulatory deficit despite improved dysarthria. The rehabilitative goals focus on further improving dynamic balance, fine motor coordination, walking efficiency and safety, communication abilities, and daily living strategies, with long-term objectives of maintaining ADL autonomy and enhancing social participation and quality of life. The intervention plan includes occupational therapy for dysarthria, ADL training, compensatory strategies for motor difficulties, and physiotherapy for balance and coordination exercises, with a good functional prognosis considering the patient’s age and observed improvements.

### Therapeutic Intervention

The therapeutic strategy for the patient, was multi-faceted, addressing both presumed autoimmune responses and the underlying metabolic syndrome that had been part of the patient’s complex medical history.

The central component of the immunomodulatory treatment was steroid therapy. Initially, a high-dose steroid regimen was administered intravenously, beginning with a bolus of prednisone at a dose reflective of the patient’s severity of symptoms and clinical judgment. Following the acute phase, the patient was transitioned to an oral tapering course. The exact dosages were as follows: 50 mg of Deltacortene, starting with two tablets at 8 AM, reducing to one tablet at 8 AM after a week, and then half a tablet in the morning until cessation at the end of the tapering period.

Concomitantly, to address the patient’s known metabolic derangements and provide supportive care, a cocktail of vitamin supplements was introduced. This included vitamin E and the B-complex vitamins, specifically, Benadon (pyridoxine) to potentially mitigate neurotoxicity and support nerve health, and Folina (folate) to counter the patient’s hyperhomocysteinemia. Neuraben (thiamine) was prescribed to support neurological function.

Additional pharmacotherapy included anticoagulant prophylaxis with Inhixa (enoxaparin) to mitigate the heightened risk of thrombotic events in the context of COVID-19 and the patient’s immobility. The patient also received Lansoprazole to guard against potential steroid-induced gastric irritation.

The patient’s clinical status remained stable throughout the treatment course. Although improvement in ataxia and dysarthria was noted, it was not profound, prompting consideration for more aggressive immunomodulatory therapy, such as intravenous immunoglobulin (IVIG), pending the results of monoclonal antibody therapy for SARS-CoV-2.

### The Patient’s Treatment Regimen

was comprehensive and designed to manage both the acute neurological symptoms and the patient’s underlying metabolic condition. The steroid therapy began with an intravenous corticosteroid treatment, followed by a tapering oral regimen. The oral steroid course consisted of Lansoprazole 30 mg once daily before breakfast to protect the gastric mucosa during steroid treatment, Deltacortene (prednisone) 25 mg starting with two tablets daily in the morning, which was then tapered down as per the schedule, and supporting medications including Vitamin E, Neuraben (thiamine), Benadon (pyridoxine), and Folina (folic acid) for neurological support and to manage metabolic issues. Inhixa (enoxaparin) was administered subcutaneously for thromboprophylaxis, and isotonic saline solution was provided intravenously twice daily. These medications aimed to reduce inflammation and support the patient’s neurological function while also addressing metabolic derangements and preventing potential complications related to both the disease process and the treatment. The response to this regimen was continuously monitored with considerations for adjusting the treatment based on the patient’s evolving clinical status and additional laboratory findings.

The patient underwent a structured rehabilitation program tailored to address the specific impairments caused by post-COVID cerebellitis. This comprehensive approach included:

- Intensive Physiotherapy: Focused on enhancing balance and coordination through specialized exercises and innovative technologies like virtual reality, which simulated real-life scenarios for improved walking safety and efficiency.
- Occupational Therapy: Aimed at boosting independence in daily activities, utilizing adaptive tools and environmental modifications to ease the transition back into home and work life.
- Speech Therapy: Targeted dysarthria improvement with exercises designed to enhance oral muscle control and speech clarity, alongside strategies to bolster effective communication.
- Cognitive Rehabilitation: Addressed potential memory and attention deficits with cognitive stimulation exercises and compensatory strategies, tailored to the patient’s specific cognitive challenges.
- Psychological Support: Provided to help the patient navigate the emotional challenges associated with their condition and recovery process, focusing on managing anxiety, depression, or stress effectively.

This personalized and adaptive rehabilitation plan played a pivotal role in the patient’s recovery, significantly improving their quality of life and facilitating a return to daily and social activities.

The patient’s rehabilitation program was intensified to include treatments twice daily, focusing on each aspect of their recovery plan. This rigorous schedule allowed for more consistent engagement in physiotherapy, occupational therapy, speech therapy, and cognitive exercises, ensuring a more continuous and impactful rehabilitation process. The twice-daily sessions facilitated a faster progression in regaining balance, coordination, independence in daily activities, speech clarity, and cognitive functions, significantly contributing to the patient’s overall improvement and return to normalcy.

### Follow-up and Outcomes

Upon admission for rehabilitation, the patient exhibited significant ataxia and dysarthria. Functional assessments revealed a Modified Barthel Index (MBI) [15] of 65/100, indicating partial autonomy in daily activities, and a Functional Ambulation Classification (FAC) [16] score of 2/5, reflecting considerable assistance needed for walking. The Trunk Control Test scored [17] 100/100, suggesting good seated trunk control, yet mobility was severely impacted by ataxia, particularly on the right side.

The rehabilitation program aimed to restore autonomy in standing (orthostasis), enhance both static and dynamic balance, regain walking independence, and improve ADL performance. Techniques included prolonged standing exercises, balance exercises with varying base support, and reduced assistance during ambulation, alongside ADL training and endurance retraining using technological gym equipment.

By discharge, the patient demonstrated remarkable improvement: the MBI improved to 91/100, indicating increased independence in daily activities. The Unified Balance Scale score rose to 70%, significantly reducing fall risk to 14 ± 5%. The FAC score improved to 4/5, allowing for near-independent walking over long distances, though fatigue revealed an extensor pattern in the right lower limb. A slight ataxic tremor in the right hand was noted during fine motor tasks, with a Time Up and Go (TUG) test further quantifying mobility enhancement. The patient was trained to perform home exercises to continue improving dynamic balance.

#### Patient Compliance

The patient demonstrated excellent compliance with the prescribed rehabilitation program, which was crucial for the significant improvements observed in their condition. This adherence to the twice-daily therapy sessions across the various disciplines of physiotherapy, occupational therapy, and speech therapy facilitated marked progress in motor and speech capabilities. The dedication to following the therapeutic interventions, including home exercises for dynamic balance improvement, played a pivotal role in the patient’s recovery journey from post-COVID cerebellitis.

## Discussion

The presented case report highlights a rare yet significant neurological complication associated with COVID-19 infection: cerebellitis. Cerebellitis, characterized by inflammation of the cerebellum, can lead to various neurological symptoms, including ataxia, dysarthria, and postural instability, as observed in the patient described [10]. The association between COVID-19 and cerebellitis remains relatively undefined, with limited evidence available primarily from case reports and small case series [2, 10, 18]. However, the growing recognition of neurological manifestations in COVID-19 patients underscores the importance of understanding and managing such complications effectively.

In this case, the patient presented with acute onset ataxia and dysarthria amid a SARS-CoV-2 infection, suggesting a likely parainfectious cerebellitis. Diagnostic evaluations, including brain imaging, ruled out other potential causes, confirming the diagnosis of cerebellitis. Other cases of COVID-19-associated cerebellitis have been reported in the literature, reinforcing the possible association between the two conditions [9, 19]. The treatment approach involved a combination of steroid therapy, vitamin supplementation, physiotherapy [12, 20–25], and intravenous immunoglobulins, which led to clinical stabilization and eventual improvement in symptoms.

The case provides critical insights into the management and pathophysiology of COVID-19-associated acute cerebellitis. Immune-mediated therapy, notably intravenous immunoglobulin (IVIg) and high-dose steroids, formed the cornerstone of the treatment regimen [18]. The rationale for this approach is supported by the immunomodulatory potential of IVIg and steroids to dampen an aberrant autoimmune response, which is often implicated in post-infectious cerebellitis. It is noteworthy that IVIg typically exhibits a latency in onset of action, requiring approximately 2–4 weeks for full therapeutic effect, with the peak impact observed around 20 days post-infusion. This timeframe is crucial for clinicians to anticipate the therapeutic trajectory and manage patient and family expectations accordingly.

The association of COVID-19 with acute cerebellitis raises the possibility of a unique immune-mediated mechanism. Evidence suggests the presence of autoantibodies against glutamate receptors, with reports such as those indicated [26], implying a direct pathogenic role. These glutamate receptor autoantibodies could alter neuronal signaling, leading to the cerebellar dysfunction observed in cerebellitis. Furthermore, other autoantibodies, including those against glutamic acid decarboxylase (GAD), have been identified in similar contexts [27], suggesting a broader autoimmune profile in COVID-19 neurological sequelae. Recent literature has identified several proposed mechanisms for central nervous system manifestations post-COVID, including cerebellitis. These include direct inflammation, where the SARS-CoV-2 virus might directly infect the cerebellum causing inflammation and neuronal damage; an aberrant immune response to the virus potentially harming cerebellar tissue; and thrombotic microangiopathy, as COVID-19 is associated with an increased risk of thrombosis which could affect cerebral vessels and lead to cerebellar damage [28].

The rehabilitation process played a crucial role in the patient’s recovery, addressing the functional limitations resulting from ataxia and dysarthria. Intensive physiotherapy focused on enhancing balance and coordination, while occupational therapy aimed at improving independence in daily activities [23, 29]. Speech therapy targeted dysarthria improvement, and cognitive rehabilitation addressed potential cognitive deficits. Psychological support was also provided to assist the patient in coping with the emotional challenges associated with their condition and recovery. Rehabilitation after cerebellitis has been the subject of various previous studies, which have highlighted the importance of a personalized rehabilitation approach to improve motor functions and patient autonomy. The inclusion of techniques such as intensive physiotherapy, occupational therapy, and speech therapy has been shown to be effective in the functional recovery of patients [30].

Significant improvements were observed in the patient’s functional abilities, as evidenced by the increased Modified Barthel Index score and improved ambulation. Despite persistent mild symptoms, such as a slight ataxic tremor, the patient demonstrated remarkable progress, highlighting the effectiveness of the multidisciplinary rehabilitation approach.

The patient’s compliance with the prescribed rehabilitation program was crucial for the observed improvements. Adherence to therapy sessions, both in the clinical setting and at home, facilitated consistent progress and contributed to the patient’s overall recovery.

Overall, this case underscores the importance of recognizing and managing neurological complications, such as cerebellitis, in COVID-19 patients. A multidisciplinary approach involving neurology, infectious diseases, rehabilitation medicine, and allied health professionals is essential for optimizing patient outcomes. Further research is needed to better understand the pathogenesis, prevalence, and optimal management strategies for neurological complications associated with COVID-19.

This case report, while providing valuable insights into neurological manifestations associated with COVID-19, comes with several limitations that affect the interpretation and generalizability of the findings. Firstly, it is based on a single patient’s experience, which limits the ability to generalize these findings to a broader population. Secondly, although the relationship between COVID-19 and cerebellitis is suggested, a definitive link cannot be established without larger, controlled epidemiological studies. Furthermore, the direct causation by SARS-CoV-2 in the cerebellar inflammation remains presumptive due to the absence of viral identification in the cerebellar tissue. Lastly, the specific impacts of the various treatments administered (steroids, IVIG, physiotherapy) on the patient’s recovery remain unclear due to the multifaceted nature of the clinical management. This necessitates further research with a larger cohort to establish more robust protocols and understand the long-term outcomes of COVID-19-associated neurological complications.

## Conclusions

This case report highlights the neurological complications associated with COVID-19, particularly cerebellitis, and demonstrates the benefits of a rapid, multidisciplinary approach to treatment. The persistent mild symptoms underscore the potential for long-lasting neurological impacts, emphasizing the importance of continued research. Further studies are needed to better understand the mechanisms, outcomes, and management of COVID-19-related neurological disorders.

### Patient Perspective

The patient’s perspective in this case is crucial for understanding the impact of cerebellitis and its treatment on their life and well-being. The patient likely faced significant challenges and uncertainties throughout their journey. The sudden onset of neurological symptoms, the diagnostic process, and the initiation of treatment would have elicited a range of emotions, from anxiety to hope. Despite progress in rehabilitation, the patient may still be adjusting to residual symptoms and striving to reintegrate into daily life. Open communication with healthcare providers and support from loved ones will be essential for the patient’s ongoing well-being. To improve the evaluation of patient perspectives, it would be useful to include standardized surveys such as HCAHPS (Hospital Consumer Assessment of Healthcare Providers and Systems) in future assessments. These surveys could provide a more structured and objective picture of patient experiences.

## Data Availability

No datasets were generated or analysed during the current study.
